# Wave reflection quantification analysis and personalized flow wave estimation based on the central aortic pressure waveform

**DOI:** 10.3389/fphys.2023.1097879

**Published:** 2023-02-23

**Authors:** Hongming Sun, Yang Yao, Wenyan Liu, Shuran Zhou, Shuo Du, Junyi Tan, Yin Yu, Lisheng Xu, Alberto Avolio

**Affiliations:** ^1^ College of Medicine and Biological and Information Engineering, Northeastern University, Shenyang, China; ^2^ School of Information Science and Technology, ShanghaiTech University, Shanghai, China; ^3^ Key Laboratory of Medical Image Computing, Ministry of Education, Shenyang, China; ^4^ Neusoft Research of Intelligent Healthcare Technology, Co. Ltd, Shenyang, China; ^5^ Macquarie Medical School, Faculty of Medicine, Health and Human Sciences, Macquarie University, Sydney, NSW, Australia

**Keywords:** wave reflection, wave separation analysis, personalized flow waveform, triangular flow waveform, arterial stiffness

## Abstract

Pulse wave reflections reflect cardiac afterload and perfusion, which yield valid indicators for monitoring cardiovascular status. Accurate quantification of pressure wave reflections requires the measurement of aortic flow wave. However, direct flow measurement involves extra equipment and well-trained operator. In this study, the personalized aortic flow waveform was estimated from the individual central aortic pressure waveform (CAPW) based on pressure-flow relations. The separated forward and backward pressure waves were used to calculate wave reflection indices such as reflection index (RI) and reflection magnitude (RM), as well as the central aortic pulse transit time (PTT). The effectiveness and feasibility of the method were validated by a set of clinical data (13 participants) and the Nektar1D Pulse Wave Database (4,374 subjects). The performance of the proposed personalized flow waveform method was compared with the traditional triangular flow waveform method and the recently proposed lognormal flow waveform method by statistical analyses. Results show that the root mean square error calculated by the personalized flow waveform approach is smaller than that of the typical triangular and lognormal flow methods, and the correlation coefficient with the measured flow waveform is higher. The estimated personalized flow waveform based on the characteristics of the CAPW can estimate wave reflection indices more accurately than the other two methods. The proposed personalized flow waveform method can be potentially used as a convenient alternative for the measurement of aortic flow waveform.

## 1 Introduction

The central aortic pressure waveform (CAPW) contains information on the cardiovascular system and thus can be used to evaluate the cardiovascular system status and to predict and diagnose cardiovascular diseases (CVDs) (Suleman et al., 2017; Vallée et al., 2018; Sequi-Dominguez et al., 2020; Flores Geronimo et al., 2021). Central aortic pressure, unlike peripheral arterial pressure, is the blood pressure at the root of the ascending aorta, which is directly connected with the left ventricle (Pini et al., 2008). Hence, CAPW can more directly reflect the load on the left ventricle, coronary arteries, and cerebral vessels and more accurately predict the occurrence of cardiovascular events and damage of target organs in comparison with the peripheral arterial pressure waveform (Roman et al., 2007; McEniery et al., 2008; Zócalo and Bia, 2022). The separation analysis of CAPW can be used to predict cardiovascular events such as all-cause mortality and left ventricular failure (Manisty et al., 2010), which is more clinically significant.

When the heart pumps blood, the aortic valve opens, and the pressure in the aorta rises rapidly, resulting in pressure and flow waves called forward waves. Forward waves will undergo wave reflections at sites of impedance mismatch (vessel diameter reduction, vessel bifurcation or change in wall stiffness) during the propagation from the aorta to the distal segments, generating backward waves, and propagating back to the proximal segment (Westerhof et al., 1972; Yao et al., 2022). When the left ventricle contracts, blood flows through the aortic valve into the aorta. After the aortic valve closes, the ventricle enters diastole, when blood perfuses the heart through the coronary arteries. A small amount of diastolic blood occasionally flows backwards into the left ventricle (Thubrikar et al., 1979). The pressure and flow waveforms are formed by the superposition of backward and forward components. Pulse wave propagation and reflection are related to arteriosclerosis and also affect the hemodynamic characteristics of the cardiovascular system (Sofogianni and Tziomalos, 2019). In pulse wave analysis, pulse wave reflection indices can be derived from the decomposition of CAPW to quantify the degree of pulse wave reflections (Townsend et al., 2015). Based on the pressure-flow relations, the CAPW can be decomposed into backward (P_b_) and forward (P_f_) waves (Westerhof et al., 1972). The amplitude characteristics and time delay of P_f_ and P_b_ can effectively reflect the reflection and propagation time of the pulse wave from the aorta to the distal segments and branches, and the magnitude of the CAPW reflections affects cardiac afterload and perfusion (Davis et al., 2009; Laurent and Boutouyrie, 2020). More accurate wave reflection measurements can be obtained from P_f_ and P_b_, mainly including the aortic pulse transit time (PTT), reflection index (RI), and reflection magnitude (RM). PTT can be calculated from the time delay between P_f_ and P_b_, a valuable indicator for assessing arterial stiffness (Qasem and Avolio, 2008). RM, the ratio of P_b_ and P_f_ amplitudes, is an independent predictor of risk and can predict heart failure (Westerhof et al., 2006; Zamani et al., 2014). RI and RM contain physiological information about CAPW and are important indices that quantify pulse wave reflection. These metrics are not affected by timing of wave reflection and usually be used to access left ventricle afterload, which has clear physiological significance (Wang et al., 2010; Zamani et al., 2016).

Flow waveforms are essential for the decomposition and analysis of pulse waves. Clinically, the aortic flow velocity can usually be obtained directly and non-invasively by ultrasonic detection or magnetic resonance imaging (MRI). Combined with the cross-sectional area of blood vessels, the blood flow waveform can finally be calculated (Rivera et al., 2020; Stortz et al., 2020). Although this method is feasible and accurate, the operation is considered inconvenient because it requires specific types of equipment and skilled operators. Consequently, some proposed approaches use the CAPW morphology to generate an aortic flow waveform with an assumed triangular shape (Westerhof et al., 2006; Butlin and Qasem, 2016). In these methods, the wave separation analysis matches the start, peak, and end points of the triangular flow waveform with the foot, inflection, and dicrotic notch points of the CAPW using the time and amplitude characteristics of the CAPW. The triangular flow wave was first proposed in a proof-of-principle study to quantify aortic wave reflections from pressure alone by Westerhof et al. (Westerhof et al., 2006). This straightforward technique was utilized by the SphygmoCor MM3/CvMS system (AtCor Medical, Sydney, Australia) for the non-invasive acquisition of aortic flow (Ding et al., 2013; Carlsen et al., 2016; Yu et al., 2018). Later, they made improvements in the waveform decomposition of the CAPW by utilizing the triangular flow waveform as a novel way for determining the aortic pulse wave velocity (Qasem and Avolio, 2008). Although triangular flow waveform has been applied in several commercially available systems, this method poorly approximates the measured flow waveform, resulting in some errors in the decomposition of the CAPW.

Kip et al. demonstrated that in the participants of the Asklepios population study, the results for RM and aortic PTT based on the triangular flow waveform approximation method differed significantly from the values obtained from measured pressure and flow information (Verbeke et al., 2005; Rietzschel et al., 2007; Kips et al., 2009). In the Asklepios population study (Rietzschel et al., 2007), the measured flow waveforms were averaged and normalized to obtain more physiological aortic flow waveforms. The experimental results have demonstrated that the average flow method can evaluate RM better than triangular flow. However, there is still a significant deviation between the approximate and the actual values. This physiological flow method has been used to assess wave reflection indices in the multi-ethnic study of atherosclerosis (Zamani et al., 2015; Zamani et al., 2016). In this research, the pressure measured non-invasively by applanation tonometry at the common carotid artery was used as a substitute for central aortic pressure. Consequently, the difference persists and influences the experimental results.

Recently, Shenouda et al. proposed a new personalized physiological flow waveform method based on the CAPW morphology (Shenouda et al., 2021). The physiological flow waveform is more accurate than the triangle flow waveform for determining RM and Pb in the elderly. However, they did not examine children, healthy middle-aged individuals, or clinical populations such as cardiac disease patients. The sample set included only 49 young (18–42 years) and 29 older (51–77 years) adults. More recently, a novel lognormal flow wave method for separating the CAPW was proposed by Hao et al. (Hao et al., 2022). This study demonstrated that the lognormal flow wave improves CAPW separation analysis results both in time and frequency domains. Nevertheless, the lognormal flow waveform method must be compared in different populations and not limited to healthy and young participants. For the data set validated in this paper, there is still a gap between the estimated and the measured flow waveforms. In addition, the definition of variance σ of the lognormal function needs to be clarified, and how to determine the specific value is not well described. When accurate flow is inconvenient to measure, better non-invasive estimation of aortic flow is still needed to improve the results of pulse wave separation of the CAPW.

This research aims to propose a novel method to approximate the actual flow waveform with a personalized flow waveform and to examine the feasibility to decompose the CAPW and quantify wave reflection. We use the relationship between pressure and flow to separate and analyze the CAPW with triangular, lognormal, and personalized flow waveform methods, respectively, to explore the accuracy of the three methods in wave reflections. Based on the simulated pulse wave dataset and clinical data, the accuracy of the personalized flow wave method is further compared with the other two methods in deducing the reflection indices of RI, RM, and PTT.

## 2 Materials and methods

### 2.1 Data collection

In this study, we used two datasets to verify the feasibility and validity of the proposed method.

#### 2.1.1 Nektar1D PWDB

The first dataset is the publicly accessible database (Nektar1D Pulse Wave Database, Nektar1D-PWDB), published by Alastruey et al. at King’s College London, United Kingdom, based on the Nektar1D model. This model used the Nektar1D non-linear one-dimensional flow model, which has been fully clinically validated and used in several studies to simulate the hemodynamic characteristics of the human arterial tree, to ensure the validity of hemodynamic parameters of the 1D model and the generated data (Matthys et al., 2007; Alastruey et al., 2011; Xiao et al., 2014; Willemet et al., 2015). For more detailed information on this database, see the study by Charlton et al. (Charlton et al., 2019).

**FIGURE 1:**
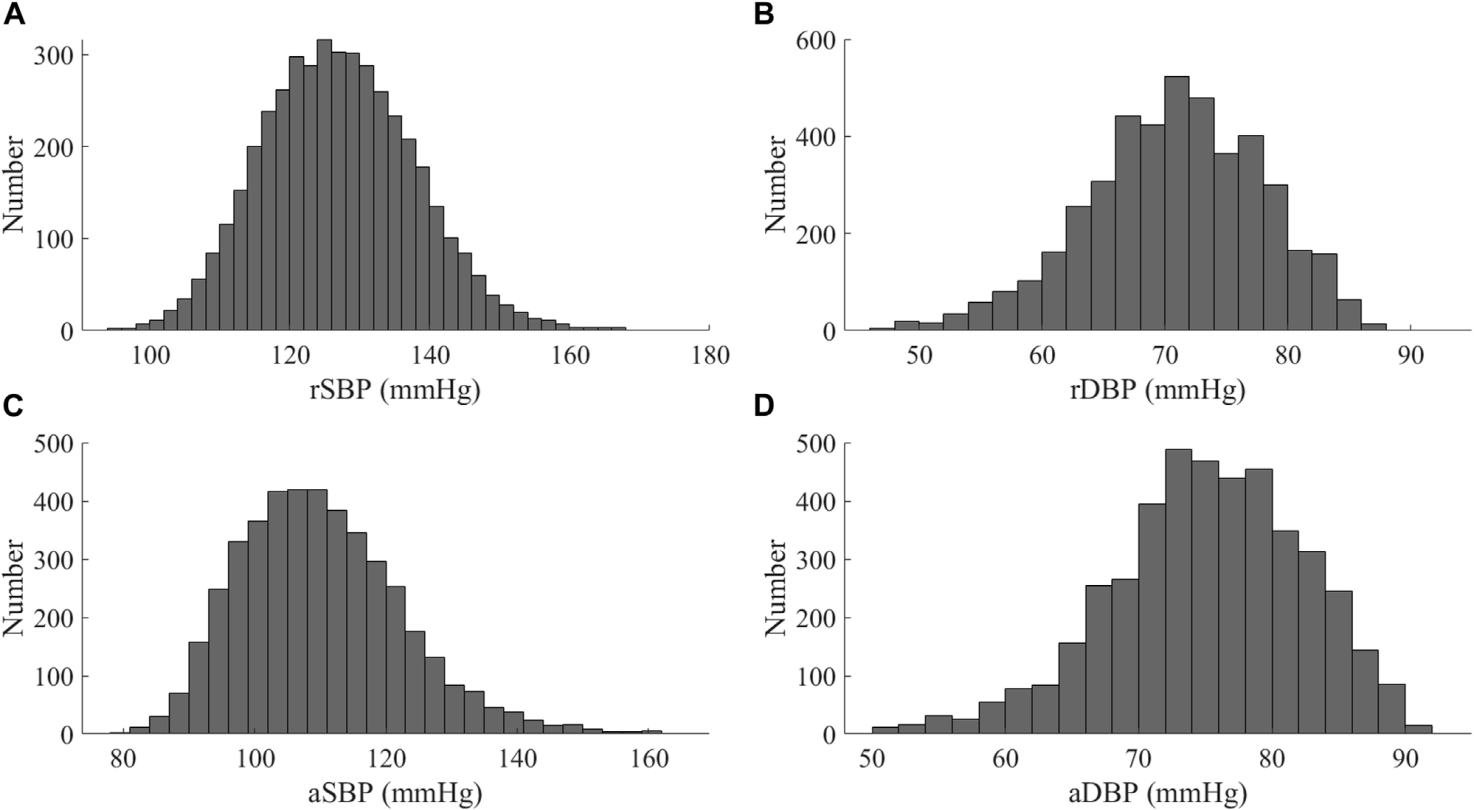
Distribution of blood pressure values for the Nektar1D-PWDB **(A)** SBP of at the radial artery (rSBP); **(B)** DBP of at the radial artery (rDBP); **(C)** SBP of at the aortic root (aSBP); **(D)** DBP of at the aortic root (aDBP).

The database contains the arterial pulse waves from 4,374 virtual subjects, ranging from 25 to 75 years, at a sampling frequency of 500 Hz. A total of 537 out of the 4,374 subjects exhibited blood pressures outside of healthy norms (virtual subjects with abnormal blood pressure; without CVD), and 3,837 subjects are physiologically plausible. Table 1 contains basic population and hemodynamic statistics. SBP and DBP of the radial artery and central aortic are 95 mmHg–168 mmHg and 48 mmHg–87 mmHg, as shown in Figure 1.

**TABLE 1:** The hemodynamic characteristics of the Nektar1D-PWDB and clinical data for all subjects. Shown as mean ± standard deviation (Mean ± SD).

Variables	Nektar1D-PWDB	Clinical data
No. Of subjects	4,374	13
Age (years)	25–75	24–33
Aortic SBP (mmHg)	109.04 ± 11.58	103.52 ± 5.86
Aortic DBP (mmHg)	75.62 ± 6.74	80.24 ± 5.72
Aortic MAP (mmHg)	86.76 ± 5.98	86.66 ± 5.93

#### 2.1.2 Clinical data

In this section, we used clinical data to further validate the performance of personalized flow waves. There were 13 healthy participants in the study, seven male and six female, aged from 24 to 33 years old. The basic information of participants is summarized in Table 1. The Research Ethics Committee approved this study of Northeastern University (NO. NEU-EC-2021B022S), China, and all participants gave informed consent.

Each participant sat quietly and relaxed for 10 min in a quiet room before measuring their brachial systolic (SBP) and diastolic (DBP) blood pressures with the Yuwell Mercury sphygmomanometer (measurement accuracy of 2 mmHg). The pressure waveforms of the radial artery were measured non-invasively with the SphygmoCor device at a sampling rate of 128 Hz. In the SphygmoCor device, the corresponding CAPW was reconstructed using a generalized radial-to-aortic transfer function. The generalized transfer function (GTF) is the most widely used method to estimate the CAPW (Sharman et al., 2006), which is obtained by simultaneous measurement of aortic and peripheral pressure (Karamanoglu et al., 1993) to obtain the corresponding function between peripheral arterial pressure and central arterial pressure, then collecting new test samples, and validating the peripheral arterial pressure waveform signal by the trained transfer function to estimate the corresponding CAPW(Cameron et al., 1998; Payne et al., 2007). The corresponding CAPW is estimated by verifying the signal of the peripheral arterial pressure waveform with the trained transfer function. The flow velocity and diameter waveforms of the aortic root were concurrently captured and smoothed by a GE Vivid E95 US system. Flow waveforms were calculated by multiplying flow velocity waveforms with the aorta’s cross-sectional area ( π× (diameter/2)^**2**^). In the study of Zhou et al., the specifics of data collection are presented (Zhou et al., 2022).

### 2.2 Wave separation analysis and wave reflection

**FIGURE 2:**
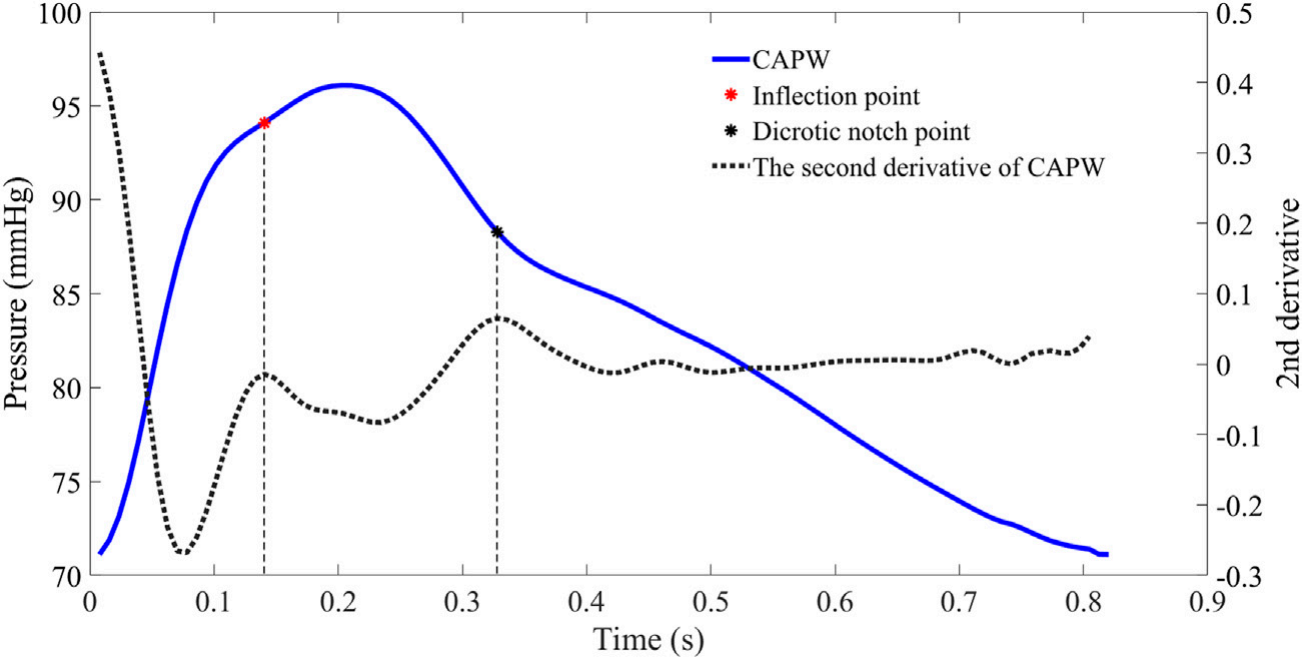
The CAPW feature points extraction. The red point represents the inflection point of the pulse wave (the moment when the P_b_ is generated), while the black point represents the dicrotic notch point (the end of the systolic phase or the beginning of the diastolic phase).

In the time domain, features can be calculated from the timing and amplitude of several fiducial points. The starting point of the pulse wave indicates the beginning of a pulse cycle and the end of the previous one. The time of the inflection point marks the arrival of the P_b_ (O'Rourke and Yaginuma, 1984). The notch is caused by aortic valve closure and blood reflux, representing the transition between the systolic and diastolic phases (Hartmann et al., 2019). The pulse wave systolic period is the duration between the starting point and the dicrotic notch point of the pulse wave, followed by the pulse wave diastolic period. Usually, the local maxima of the second derivative of the pulse waveforms are utilized to extract inflection points and dicrotic notch points (as in Figure 2 (Vlachopoulos et al., 2011)).

**FIGURE 3:**
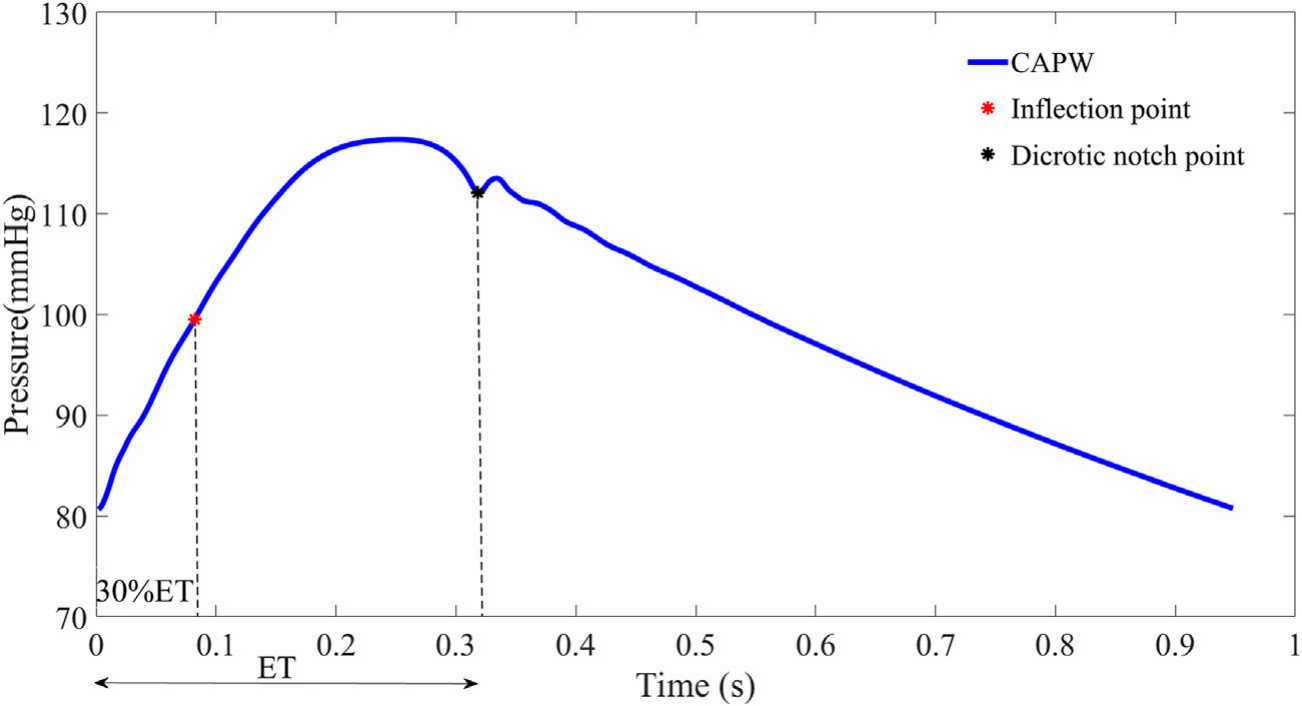
To facilitate wave separation analysis, the 30% ET is used as the location of the inflection point of the pulse wave.

For some participants (e.g., those with severe atherosclerosis), the inflection point of the aortic pulse wave is difficult or even impossible to extract. In order to make this pulse wave decomposition method more practical, it has been proposed to use 30% of the systolic time as the location of the inflection point (Miyashita et al., 1994; Westerhof et al., 2006). In this paper, for pulse wave with inconspicuous inflection point, 30% of ejection time (ET) is used as the location of the inflection point to calculate the relevant features of pulse wave decomposition. The beginning of the pulse wave systole indicates the time of aortic valve opening and the start of ejection, and the notch time of the pulse wave is the time of aortic valve closure and the end of ejection. ET represents the systolic time of the pulse wave, which is determined by subtracting the beginning time from the end time of aortic flow (as in Figure 3).

**FIGURE 4:**
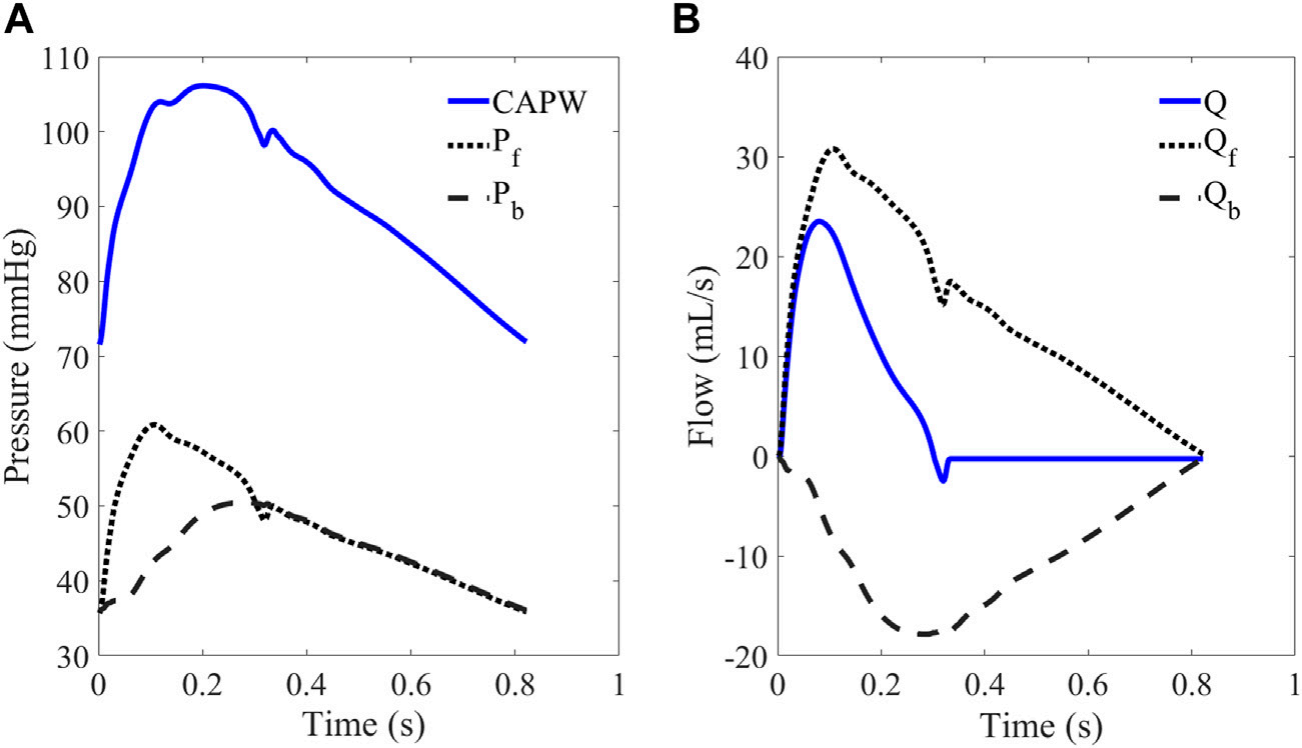
**(A)** CAPW and **(B)** flow waveform. The CAPW is decomposed into P_f_ and P_b_, from which RM and RI can be calculated.

In the arterial system, both aortic pressure and flow waveforms consist of forward waves (P_f_, Q_f_) and backward waves (P_b_, Q_b_). The CAPW mainly comprises forward and lower limb reflection waves (Westerhof et al., 1972). As shown in Figure 4, CAPW equals the sum of the P_f_ and P_b_; and the flow wave equals the difference between the Q_f_ and Q_b_, (as shown in Eq. 1, 2).

$$P=P_{f}+P_{b}$$ (1)

$$Q=Q_{f}+Q_{b}$$ (2)

The basic principle of pulse wave decomposition is as follows (Westerhof et al., 1972):

$$P_{f}=\frac{P+Z_{c}\times Q}{2}$$ (3)

$$P_{b}=\frac{P-Z_{c}\times Q}{2}$$ (4)

where, Q **=** U*A represents aortic flow; U is the flow velocity; A is blood vessels cross-sectional area; Z_c_ is the characteristic impedance.

Since the pulse waveform is not affected by the P_b_ in the early systolic phase, Z_c_ equals the ratio of blood pressure to flow (Li, 1986; Khir et al., 2001), and Z_c_ can also be calculated by high-frequency input impedance (Murgo et al., 1981; Miyashita et al., 1994). The input impedance (Z_in_) is defined as follows:

$$Z_{in}\left(w\right)=P\left(w\right)/Q\left(w\right)$$ (5)

where P(w) and Q(w) are pressure and flow frequency components.

RI is the amplitude ratio of P_b_ to the sum of P_b_ and P_f_, and the amplitude ratio of P_b_ to P_f_ is RM (Hametner et al., 2013). RM and RI are defined as follows:

$$RM=\frac{\left|P_{b}\right|}{\left|P_{f}\right|}$$ (6)

$$RI=\frac{\left|P_{b}\right|}{\left|P_{b}\right|+\left|P_{f}\right|}$$ (7)

PTT can be determined by pulse wave decomposition, an important index to assess arterial stiffness in the young and old (Qasem and Avolio, 2008). PTT can be calculated as half the time difference between P_f_ and P_b_ (T_fb_), as in Eq. 8.

$$PTT=T_{fb}/2$$ (8)

**FIGURE 5:**
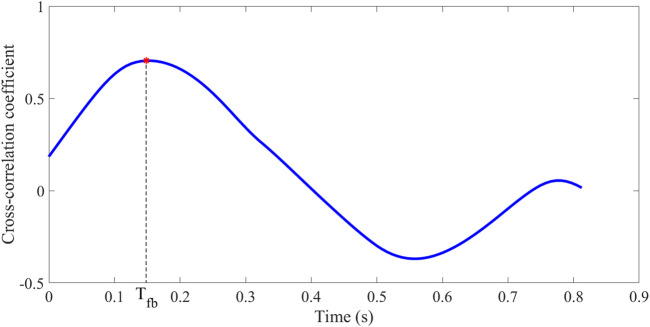
Calculation of T_fb_: cross-correlation between P_f_ and P_b_.

Qasem and Avolio calculated the cross-correlation coefficient of P_f_ and P_b_ to determine T_fb_ (Qasem and Avolio, 2008). The time of maximum cross-correlation coefficient is the T_fb_ between P_f_ and P_b_ (as in Figure 5).

#### 2.2.1 Triangular and lognormal flow waveform

By measuring aortic flow velocities with Doppler ultrasound or magnetic resonance imaging (MRI) and combining them with the cross-sectional area of the aortic valves, the aortic flow can be calculated (Wang et al., 2010; Zamani et al., 2016). However, this requires specific medical equipment and skilled operators.

**FIGURE 6:**
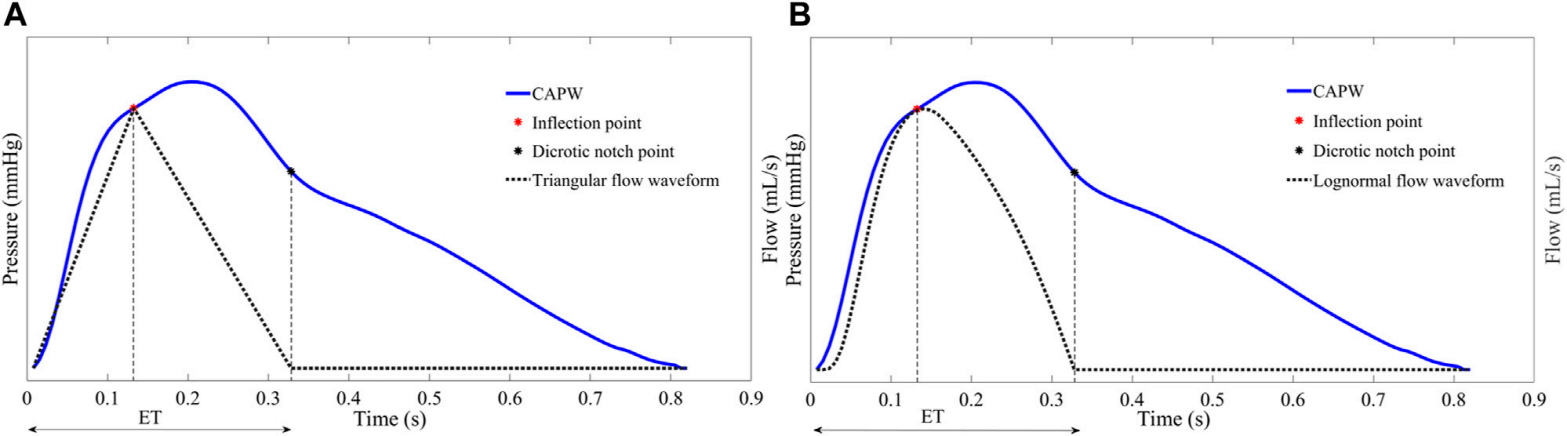
**(A)** The start, peak, and end of the triangle flow waveform correspond in time and amplitude to the foot, inflection point, and dicrotic notch point of the CAPW, respectively **(B)** Lognormal function approximation flow waveform.

The triangular flow method is used in the SphygmoCor MM3/CVMS device, which is well clinically validated and certified by the Food and Drug Administration (FDA) and is frequently used as a non-invasive testing standard to validate other devices (Zuo et al., 2010; Ott et al., 2012; Laugesen et al., 2014). SphygmoCor MM3/CVMS system uses triangles to approximate the central aortic flow waveforms (Rivera et al., 2020). Specifically, as shown in Figure 6A, the systolic flow is approximated as a triangle, and the base of the triangle represents the total systolic ET. The peak of the triangle corresponds to the inflection point (timing and amplitude) of the CAPW. Furthermore, the beginning and ending points of the triangular flow waveform coincide with the CAPW foot and dicrotic notch points, respectively. Westerhof et al. have shown that it is feasible to construct the aortic flow waveform by a triangular wave (Westerhof et al., 2006).

As with the triangular flow waveform, there is a specific relationship between the characteristic points of the lognormal flow waveform and the characteristic points of CAPW. As shown in Figure 6B, the start, peak, and end points of the lognormal flow waveform correspond to the foot, the inflection point, and the dicrotic notch point of the CAPW, respectively (Plamondon et al., 2013; Hao et al., 2022).

#### 2.2.2 Personalized flow waveform construction

The waveform of aortic flow can be estimated using a triangular wave. However, the P_f_ and P_b_ obtained directly using the triangular wave instead of the flow wave are not smooth and sometimes produce large P_b_ before the reflection point. The triangular flow waveform would also underestimate the degree of concavity of the flow waveforms. The lognormal approximate flow waveform has the same result, especially in early systole (as in Figure 8). Based on the above facts, we attempted to construct a flow waveform based on the characteristics of CAPW and explore the method’s generalizability.

In early systole (before the inflection point), the CAPW is linear with flow waveform because wave reflections are almost unaffected by the P_b_ (Hughes et al., 2020). The P_f_ propagates from the proximal to the distal end, and at the end of the contraction, the pressure-flow waves encounter a high impedance location for continuous decay. At the end of systole, it is proposed to use the Hermite interpolation function to fit the flow waveforms during this period.

The Hermite interpolation function is a standard method for solving predictive problems in mathematical modeling, which can effectively solve the problem such as insufficient waveform data of aortic flow (Lorentz, 2000). Three points are required to satisfy the Hermitian interpolation function condition. Using segmented Hermite interpolation to obtain a smooth and continuous curve on the interval $\left[a,b\right]$ . On node $a\leq x_{0}<x_{1}<\cdots <x_{n}\leq b,h_{i}=x_{i}-x_{i-1}\left(i=1,2,\cdots ,n\right)$ , the function value and derivative value of the given node are as follows:

$$y_{i}=f\left(x_{i}\right),y_{i}^{\prime }=f^{\prime }\left(x_{i}\right),i=0,1,\cdots ,n$$ (9)

A piecewise cubic interpolation polynomial $H_{3}\left(x\right)$ is constructed on $\left[a,b\right]$ , which satisfies the following interpolation conditions:

$$H_{3}\left(x_{i}\right)=y_{i},H_{3}^{\prime }\left(x_{i}\right)=y_{i}^{\prime },i=0,1,\cdots ,n$$ (10)

$H_{3}\left(x\right)$ on the interval $\left[x_{i-1},x_{i}\right]$ is the cubic Hermite interpolation polynomial of $f\left(x\right)$ with $x_{i-1},x_{i}$ as nodes.

$$\begin{array}{l} H_{3}\left(x\right)=\frac{1}{h_{i}^{2}}[\left(1+2\frac{x-x_{i-1}}{h_{i}}\right){\left(x-x_{i}\right)}^{2}y_{i-1}+\left(1-2\frac{x-x_{i}}{h_{i}}\right){\left(x-x_{i-1}\right)}^{2}y_{i}\\ \left.+\left(x-x_{i-1}\right){\left(x-x_{i}\right)}^{2}y_{i-1}^{\prime }+{\left(x-x_{i-1}\right)}^{2}\left(x-x_{i}\right)y_{i}^{\prime }\right] \end{array}$$ (11)

where $x\in \left[x_{i-1},x_{i}\right]\;\left(i=1,2,\cdots ,n\right)$ .

The process of constructing the personalized flow waveform based on CAPW features is divided into three steps.

**FIGURE 7:**
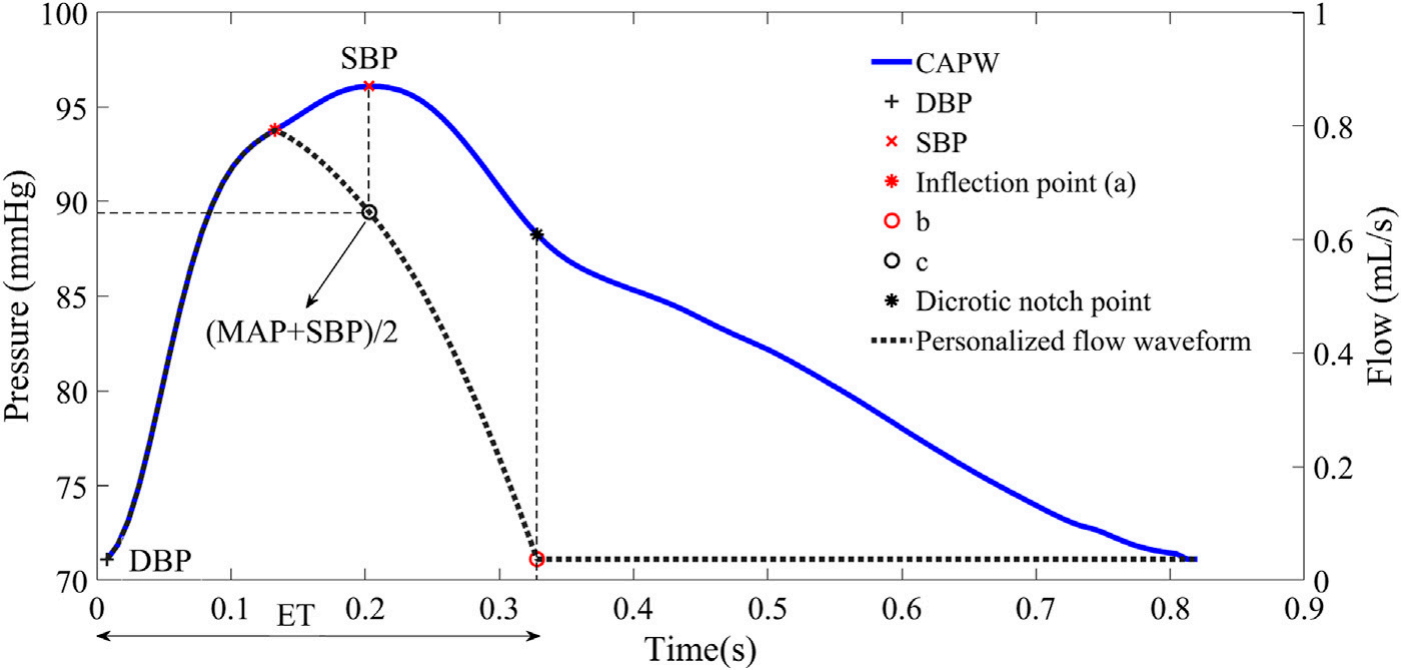
Personalized flow waveform constructed based on the feature points of CAPW.

1) The first part is the same as the CAPW before the inflection point.
2) We used the piecewise cubic Hermitian interpolation function at the end-systole to obtain the second part of the estimated flow waveform. Two points, a and b (see Figure 7), can be readily obtained, but a third point is still needed to perform the Hermite function operation. The third point was identified as c, because the magnitude of MAP and the time of SBP in CAPW are between a and b (Li et al., 2021; Parittotokkaporn et al., 2021), respectively. We combine the magnitudes of MAP and SBP and the time of SBP to obtain c for participating in the Hermitian interpolation calculation. The average value of arterial blood pressure during a cardiac cycle is called mean arterial pressure (MAP). MAP can be calculated by Eq. 12 (Papaioannou et al., 2016).

$$MAP=\frac{\int_{T}CAPW\left(t\right)\;dt}{T}$$ (12)

Where T represents a cardiac cycle. SBP and DBP are systolic and diastolic blood pressure, respectively. In the arterial system, the maximum peak and foot amplitudes of CAPW are SBP and DBP (as in Figure 7 (Avolio et al., 2009)), respectively.

3) The rest of the flow waveform is set to 0.

**FIGURE 8:**
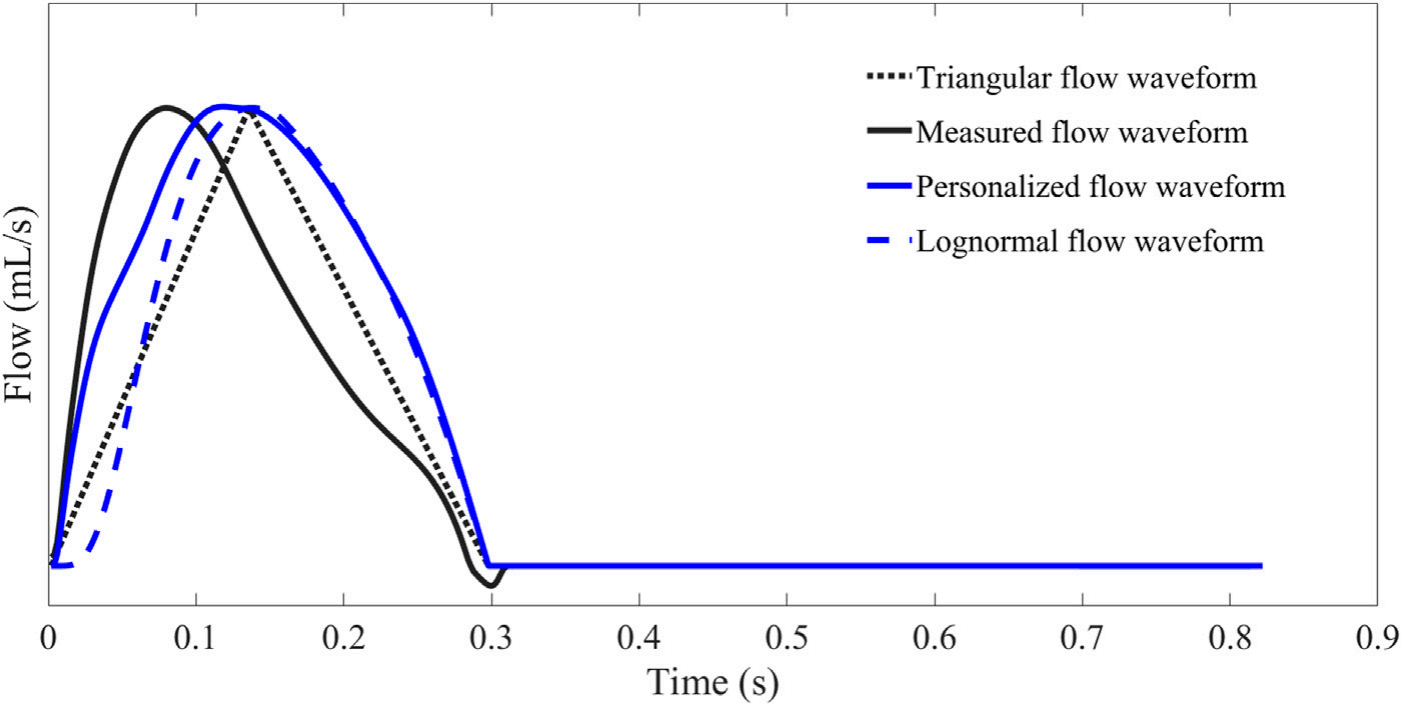
Comparison and contrast of flow waveforms obtained by Hermite interpolation function estimation, measurement, triangular estimation, and lognormal approximation.

The waveforms of personalized flow, measured flow, triangular flow, and lognormal flow approximation are shown in Figure 8. The comparison of different flow waveforms reveals a prominent peak in the triangular estimated flow waveform, which has a considerable discrepancy with the measured flow waveform. In contrast, the estimated personalized flow waveform is closer in shape to the measured flow waveform. Additionally, there are also some variations between the lognormal flow waveform and the measured flow waveform, particularly in the initial part.

To further verify the viability of the proposed personalized flow wave, the three wave reflection indices RM, RI, and PTT of wave separation analysis are quantitatively compared based on triangular flow waveform, lognormal flow wave approximation, and personalized flow waveform, respectively (Table 2). We investigated the correlation and consistency of calculated RM, RI, and PTT on the Nektar1D PWDB dataset and clinical data using linear regression analysis (r-values) and Bland-Altman analysis (see Figures 10–15), respectively.

**TABLE 2:** Wave reflection indices (Mean ± SD) and biases statistics (RMSE: Root Mean Square Error).

Database	Variable	Wave reflection indices and biases (RMSE)			
		Measured flow	Personalized flow and |Measured-Personalized|	Lognormal flow and |Measured-Lognormal|	Triangular flow and |Measured-Triangular|
**Nektar1D PWDB (n = 4,374)**	Q (mL/s)	2.83 ± 5.62	**3.04 ± 4.98**	3.12 ± 5.20	4.22 ± 6.28
		**—**	**0.89**	0.92	2.33
	P_f_ amplitude (mmHg)	21.95 ± 8.49	**22.58 ± 7.99**	22.85 ± 9.47	20.8 ± 9.41
		**—**	**1.39**	2.01	2.38
	P_b_ amplitude (mmHg)	15.8 ± 6.81	**15.02 ± 5.94**	14.99 ± 5.98	16.06 ± 7.07
		**—**	**0.39**	1.19	1.24
	RM (%)	71.49 ± 9.55	**73.84 ± 4.14**	66.15 ± 3.66	63.88 ± 9.73
		**—**	**5.88**	9.06	10.17
	RI (%)	41.5 ± 3.4	**42.27 ± 1.57**	39.78 ± 1.34	38.94 ± 3.33
		**—**	**1.95**	3.09	3.47
	PTT (ms)	34.9 ± 13.1	**37.9 ± 14.3**	28.1 ± 15.9	23.7 ± 21.4
		**—**	**1.21**	1.23	1.52
**Clinical data (n = 13)**	Q (mL/s)	5.52 ± 8.07	**5.41 ± 8.12**	5.10 ± 8.00	4.43 ± 6.92
		**—**	**2.15**	3.20	2.84
	P_f_ amplitude (mmHg)	20.36 ± 5.16	**21.39 ± 5.7**	23.91 ± 7.41	35.14 ± 14.9
		**—**	**3.29**	4.16	7.35
	P_b_ amplitude (mmHg)	9.93 ± 2.9	**10.19 ± 2.8**	10.89 ± 2.9	11.79 ± 3.4
		**—**	**1.37**	1.59	2.15
	RM (%)	88.41 ± 2.62	**87.69 ± 2.76**	87.61 ± 3.6	83.34 ± 6.66
		**—**	**1.62**	2.25	3.76
	RI (%)	48.04 ± 1.16	**48.03 ± 1.55**	48.07 ± 1.47	46.46 ± 1.94
		**—**	**0.70**	0.93	2.26
	PTT (ms)	75.4 ± 15.9	**79.5 ± 15**	80.8 ± 18.7	80.4 ± 15.8
		**—**	**0.97**	1.13	1.86

The bold values in Table 2 are the wave reflection indices results of the personalized flow wave, which have the smallest biases with the measured flow wave.

### 2.3 Evaluation and statistical analysis

In the experiment, we employed the root mean square error (RMSE) to quantitatively evaluate the deviation between measured and estimated flow waveform signals. Differences between wave reflection indices of the estimated and measured aortic flow waveforms were analyzed by two-tailed paired t tests (IBM SPSS Statistics, version-26) and reported as mean ± standard deviation (Mean ± SD) or 95% CI where appropriate. Linear regression and Pearson correlation coefficients were used to analyze the correlations between estimated and measured and aortic flow waveforms. Bland-Altman plots were constructed to assess the agreement between estimated and measured aortic flow waveforms. A *p*-value of 0.01 or less is regarded as statistically significant.

## 3 Results

### 3.1 Waveform analysis of P_f_ and P_b_

**FIGURE 9:**
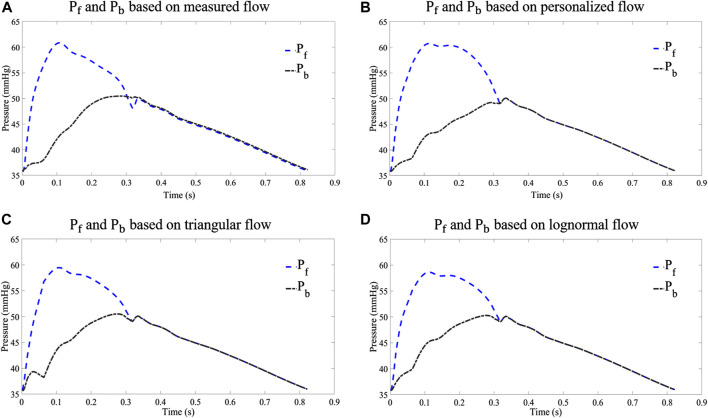
Comparison of P_f_ and P_b_ decomposed from different flow waves: **(A)** results of waveform separation based on measured flow wave; **(B)** results of waveform separation based on personalized flow wave; **(C)** results of waveform separation based on triangular flow wave; and **(D)** results of waveform separation based on lognormal flow wave approximation.

In order to analyze the performance of the flow waveform estimation using the personalized flow method, the results are compared with the typical triangular flow method and lognormal flow wave approximation. Figure 9 shows an example of the P_f_ and P_b_ decomposed by four flow waves for CAPW, respectively. The results of CAPW separation show that both P_f_ and P_b_ have different degrees of triangular wave traces when separated by the triangular flow waveform. As shown in Figure 9C, the P_b_ decomposed by the triangular flow waveform appears as a sharp peak at its foot, like the triangular flow wave’s triangular apex. However, this does not occur using personalized and lognormal flow waves, as shown in Figure 9B,D. Neither P_f_ nor P_b_ calculated by the measured flow wave in a practical situation exhibit traces of a triangle (Figure 9A). And there are no triangular features at the feet of P_f_ and P_b_. Therefore, the decomposition of CAPW using a personalized flow wave is better than the triangular flow wave analysis. The personalized flow wave performs well in estimating the morphology of P_f_ and P_b_, which is closer to the reference flow wave (Figure 9B).

### 3.2 Performance evaluation of wave reflection indices

**FIGURE 10:**
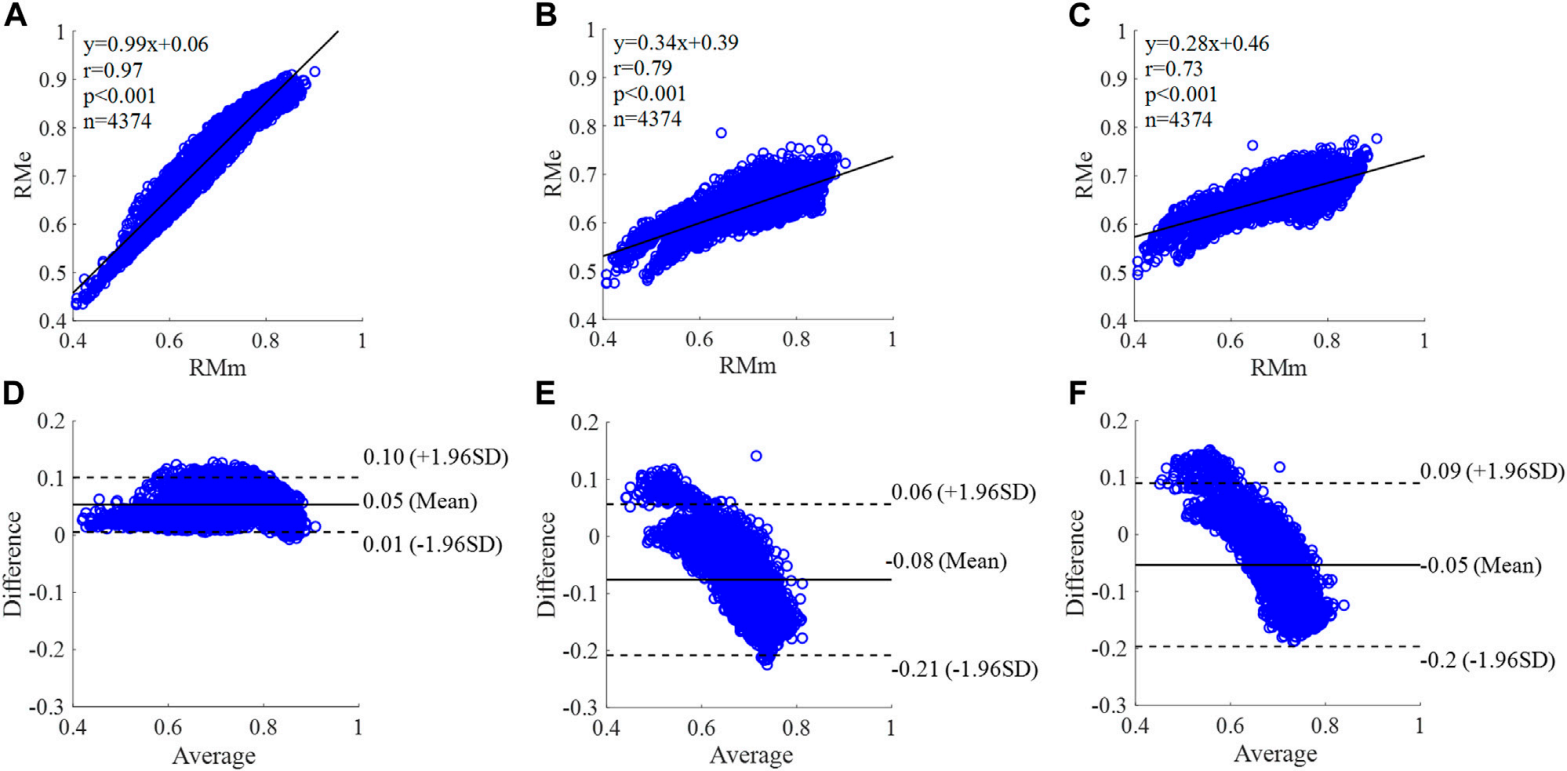
Correlation graphs and Bland-Altman plots of RM calculated by three flow waveforms **(A)** and **(D)** Results of the personalized flow wave (Nektar1D PWDB, 4,374 subjects); **(B)** and **(E)** Results of the triangular flow wave (Nektar1D PWDB, 4,374 subjects); **(C)** and **(F)** Results of the lognormal flow wave (Nektar1D PWDB, 4,374 subjects). RMm and RMe are measured and estimated RM, respectively. Difference: RMe - RMm; Average: (RMe + RMm)/2.

**FIGURE 11:**
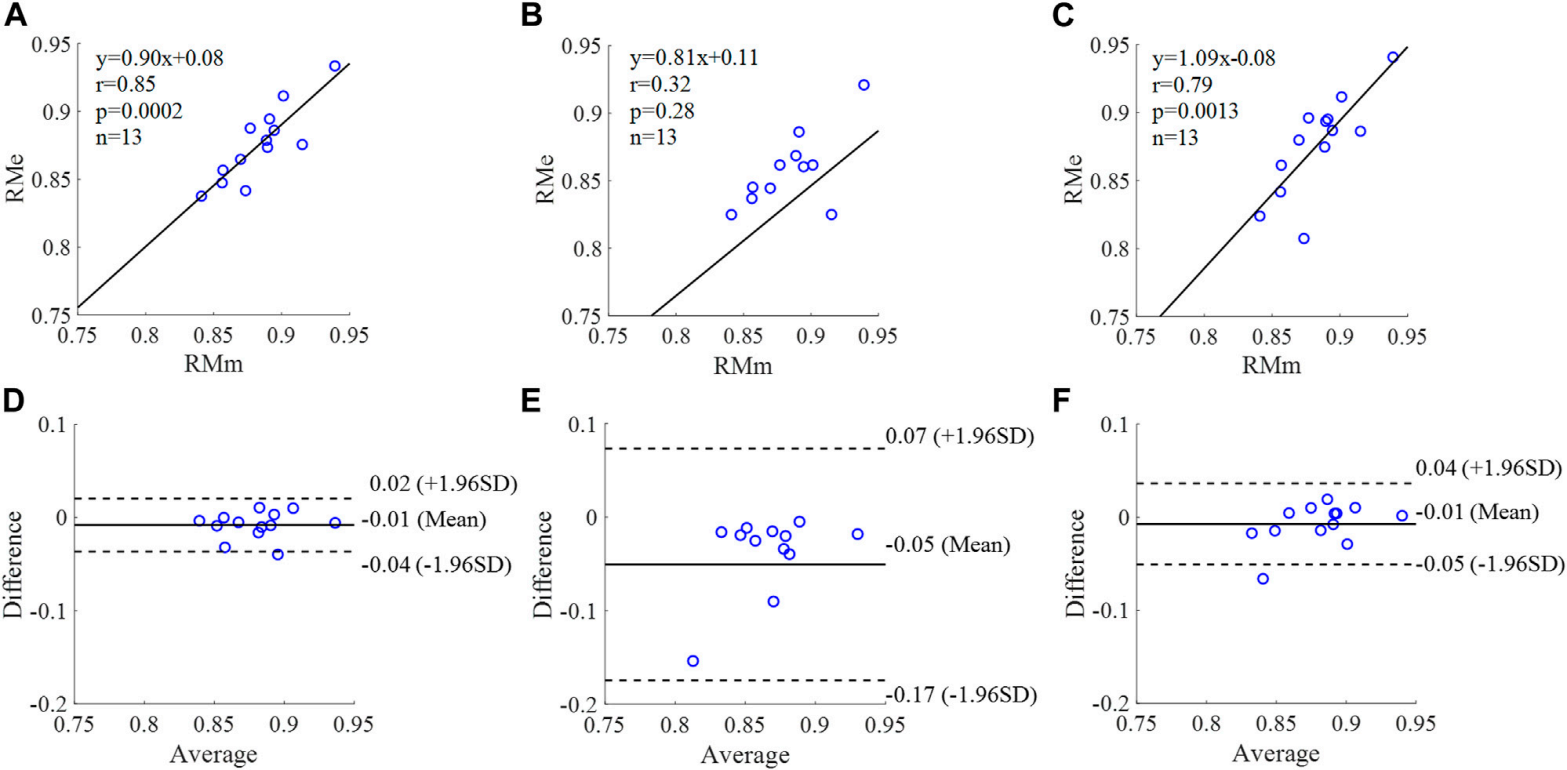
Correlation graphs and Bland-Altman plots of RM calculated by three flow waveforms **(A)** and **(D)** Results of the personalized flow wave (Clinical data, 13 participants); **(B)** and **(E)** Results of the triangular flow wave (Clinical data, 13 participants); **(C)** and **(F)** Results of the lognormal flow wave (Clinical data, 13 participants). RMm and RMe are measured and estimated RM, respectively. Difference: RMe - RMm; Average: (RMe + RMm)/2.

**FIGURE 12:**
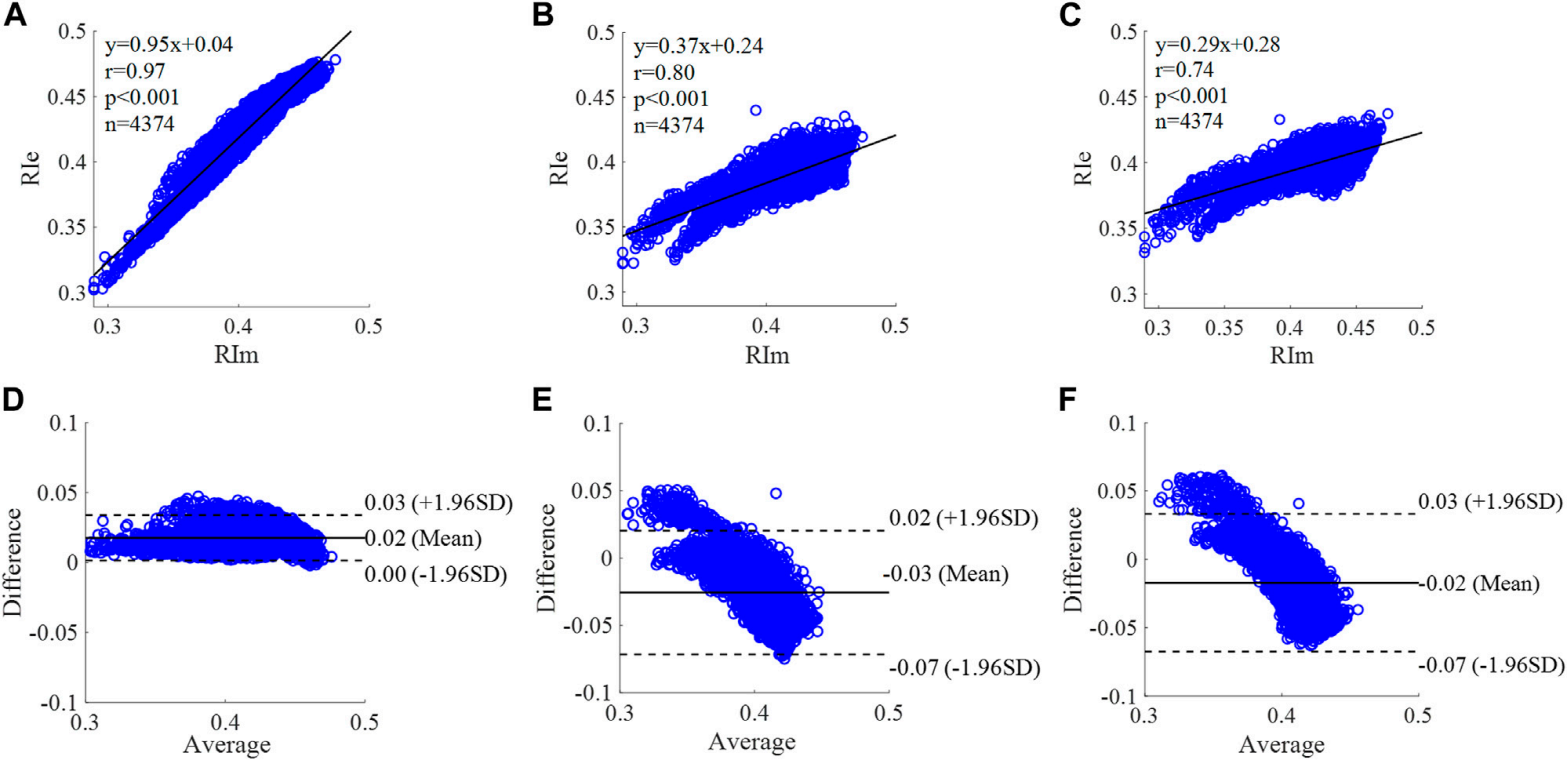
Correlation graphs and Bland-Altman plots of RI calculated by three flow waveforms. **(A)** and **(D)** Results of the personalized flow wave (Nektar1D PWDB, 4,374 subjects); **(B)** and **(E)** Results of the triangular flow wave (Nektar1D PWDB, 4,374 subjects); **(C)** and **(F)** Results of the lognormal flow wave (Nektar1D PWDB, 4,374 subjects). RIm and RIe are measured and estimated RI, respectively. Difference: RIe - RIm; Average: (RIe + RIm)/2.

**FIGURE 13:**
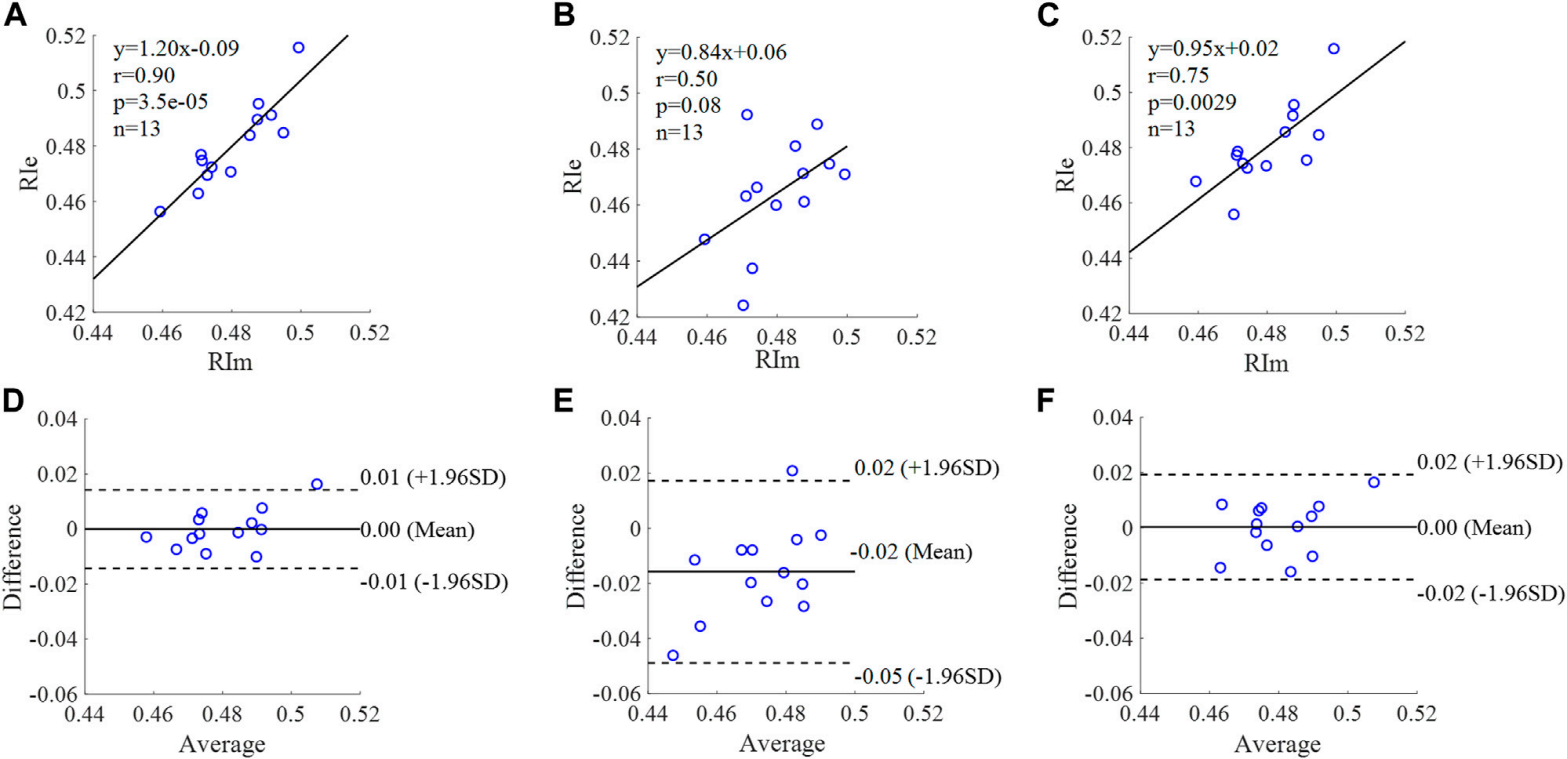
Correlation graphs and Bland-Altman plots of RI calculated by three flow waveforms. **(A)** and **(D)** Results of the personalized flow wave (Clinical data, 13 participants); **(B)** and **(E)** Results of the triangular flow wave (Clinical data, 13 participants); **(C)** and **(F)** Results of the lognormal flow wave (Clinical data, 13 participants). RIm and RIe are measured and estimated RI, respectively. Difference: RIe - RIm; Average: (RIe + RIm)/2.

**FIGURE 14:**
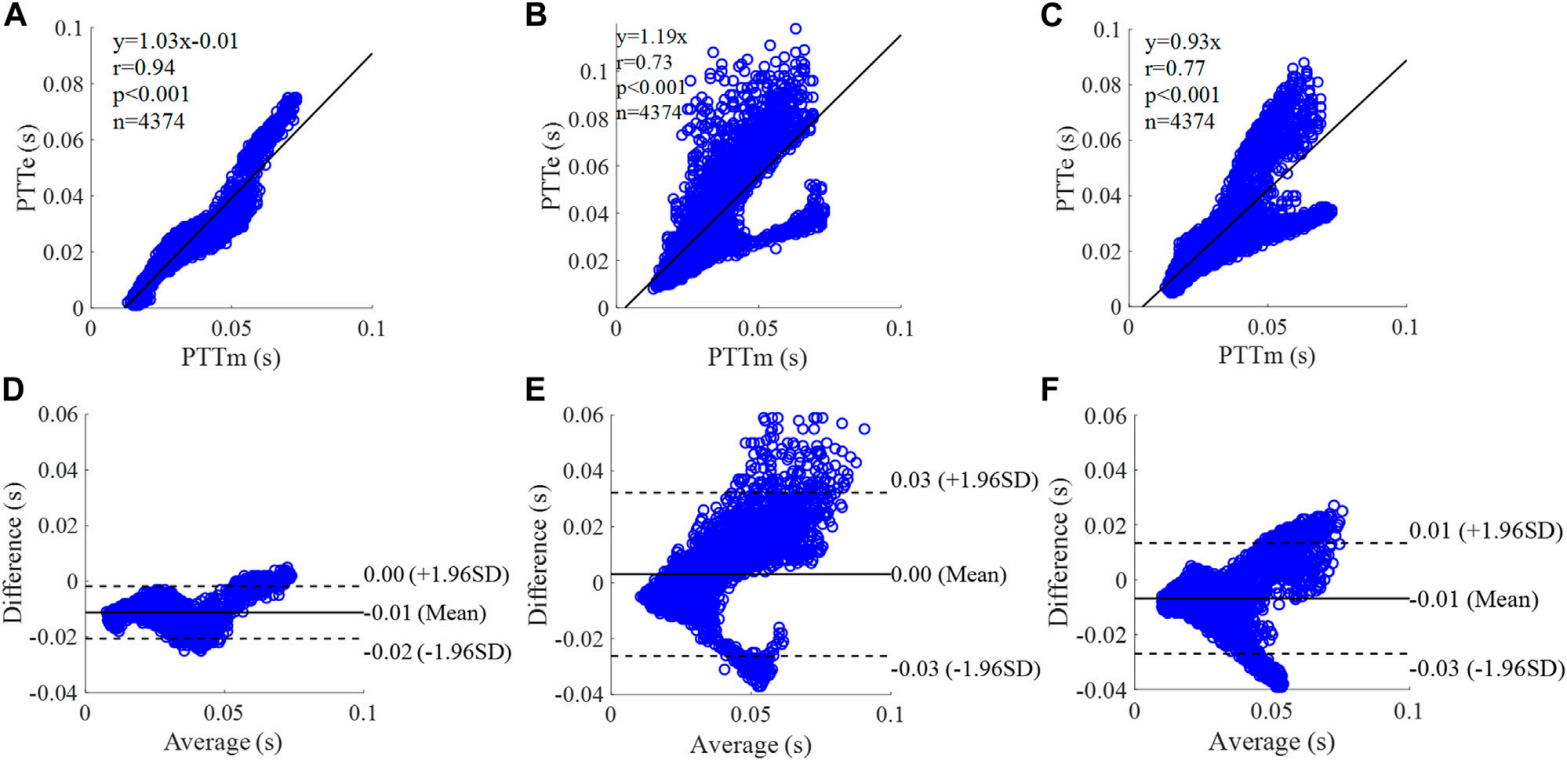
Correlation graphs and Bland-Altman plots of PTT calculated by three flow waveforms **(A)** and **(D)** Results of the personalized flow wave (Nektar1D PWDB, 4,374 subjects); **(B)** and **(E)** Results of the triangular flow wave (Nektar1D PWDB, 4,374 subjects); **(C)** and **(F)** Results of the lognormal flow wave (Nektar1D PWDB, 4,374 subjects). PTTm and PTTe are measured and estimated PTT, respectively. Difference: PTTe - PTTm; Average: (PTTe + PTTm)/2.

**FIGURE 15:**
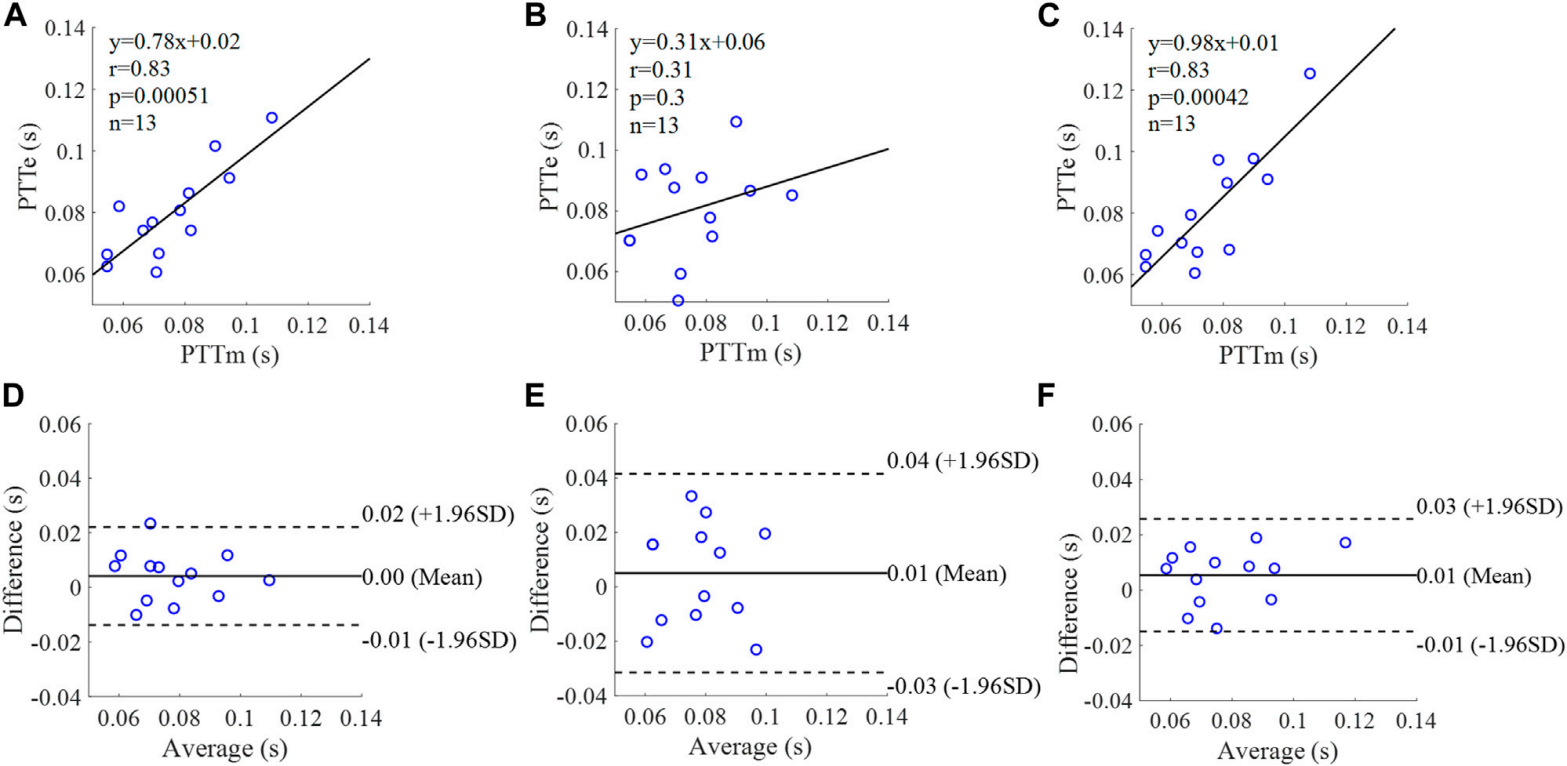
Correlation graphs and Bland-Altman plots of PTT calculated by three flow waveforms **(A)** and **(D)** Results of the personalized flow wave (Clinical data, 13 participants); **(B)** and **(E)** Results of the triangular flow wave (Clinical data, 13 participants); **(C)** and **(F)** Results of the lognormal flow wave (Clinical data, 13 participants). PTTm and PTTe are measured and estimated PTT, respectively. Difference: PTTe - PTTm; Average: (PTTe + PTTm)/2.

The corresponding correlation graphs and Bland-Altman plots for comparing measured and estimated flow CAPW reflection indices using three flow wave methods as shown in Figures 10–15.

The equation of the linear regression obtained between the measured and estimated RM using the personalized flow method based on Nektar1D PWDB is **y = 0.99x + 0.06** (**r = 0.97**, **p < 0.001**) as shown in Figure 10A; The corresponding equations obtained using the triangular flow approach and lognormal flow approximation (see Figure 10B,C) are y = 0.34x + 0.39 (r = 0.79, *p* < 0.001) and y = 0.28x +0.46 (r = 0.73, *p* < 0.001), respectively. A comparison (mean ± SD, **0.05 ± 0.03**) between the measured and estimated RM using the personalized flow method based on Nektar1D PWDB is shown in Figure 10D. The same comparison using the triangular flow approach and lognormal flow approximation (mean ± SD, −0.08 ± 0.07 and −0.05 ± 0.07) is shown in Figure 10E,F, respectively. The linear regression and Bland-Altman plots of RM calculated by three flow waveforms (Clinical data, 13 participants) are shown in Figure 11. The regression equations (panels A, B and C) are **y = 0.90x+0.08** (**r = 0.85**, **p < 0.001**), for the personalized flow wave method; y = 0.81x+0.11 (r = 0.32, *p* = 0.28) for the triangular flow wave approach; and y = 1.09x-0.08 (r = 0.79, *p* = 0.0013) for the lognormal flow wave approximation algorithm. The corresponding Bland-Altman plots (panels D, E and F) and their mean differences ( ± SD) for the personalized flow wave, triangular flow wave and lognormal flow wave methods respectively are (**− 0.01 ± 0.15**), (−0.05 ± 0.61) and (−0.01 ± 0.26).

The equation of the linear regression obtained between the measured and estimated RI using the personalized flow method based on Nektar1D PWDB is **y = 0.95x + 0.04** (**r = 0.97**, **p < 0.001**) as shown in Figure 12A; The corresponding equations obtained using the triangular flow method and lognormal flow approximation (see Figure 12B,C) are y = 0.37x + 0.24 (r = 0.80, *p* < 0.001) and y = 0.29x + 0.28 (r = 0.74, *p* < 0.001), respectively. A comparison (mean ± SD, **0.02 ± 0.01**) between the measured and estimated RI using the personalized flow method based on Nektar1D PWDB is shown in Figure 12D. The same comparison using the triangular flow method and lognormal flow approximation (mean ± SD, −0.03 ± 0.03 and −0.02 ± 0.03) is shown in Figure 12E,F, respectively. The linear regression and Bland-Altman plots of RI calculated by three flow waveforms (Clinical data, 13 participants) are shown in Figure 13. The regression equations (panels A, B and C) are **y = 1.20x-0.09** (**r = 0.90**, **p < 0.001**), for the personalized flow wave method; y = 0.84x+0.06 (r = 0.50, *p* = 0.08) for the triangular flow wave approach; and y = 0.95x+0.02 (r = 0.75, *p* = 0.0029) for the lognormal flow wave approximation algorithm. The corresponding Bland-Altman plots (panels D, E and F) and their mean differences ( ± SD) for the personalized flow wave, triangular flow wave and lognormal flow wave methods respectively are (**0 ± 0.01**), (−0.02 ± 0.02) and (0 ± 0.01).

The equation of the linear regression obtained between the measured and estimated PTT using the personalized flow method based on Nektar1D PWDB is **y = 1.03x - 0.01** (**r = 0.94**, **p < 0.001**) as shown in Figure 14A; The corresponding equations obtained using the triangular flow method and lognormal flow approximation (see Figure 14B,C) are y = 1.19x (r = 0.73, *p* < 0.001) and y = 0.93x (r = 0.77, *p* < 0.001), respectively. A comparison (mean ± SD, **-0.01 ± 0.01 s**) between the measured and estimated PTT using the personalized flow method based on Nektar1D PWDB is shown in Figure 14D. The same comparison using the triangular flow method and lognormal flow approximation (mean ± SD, 0 ± 0.02 s and −0.01 ± 0.01 s) is shown in Figure 14E,F, respectively. The linear regression and Bland-Altman plots of PTT calculated by three flow waveforms (Clinical data, 13 participants) are shown in Figure 15. The regression equations (panels A, B and C) are **y = 0.78x+0.02** (**r = 0.83**, **p < 0.001**), for the personalized flow wave method; y = 0.31x+0.06 (r = 0.31, *p* = 0.3) for the triangular flow wave approach; and y = 0.98x+0.01 (r = 0.83, *p* < 0.001) for the lognormal flow wave approximation algorithm. The corresponding Bland-Altman plots (panels D, E and F) and their mean differences ( ± SD) for the personalized flow wave, triangular flow wave and lognormal flow wave methods respectively are (**0 ± 0.01 s**), (0.01 ± 0.02 s) and (0.01 ± 0.01 s).

The coefficient of determination between the measured and estimated RM using the personalized flow method based on two datasets are **0.94** and **0.72**, and the results of using the triangular flow method are 0.62 and 0.10. The results of using the lognormal flow wave approximation are 0.53 and 0.62. The coefficient of determination between the measured and estimated RI using the personalized flow method based on two datasets are **0.94** and **0.81**, and the results of using the triangular flow method are 0.64 and 0.25. The results of using the lognormal flow wave approximation are 0.55 and 0.56. The coefficient of determination between the measured and estimated PTT using the personalized flow method based on two datasets are **0.88** and **0.69**, and the results of using the triangular flow method are 0.53 and 0.09. The results of using the lognormal flow wave approximation are 0.59 and 0.69. Therefore, the correlation of the reflection indices calculated by the personalized flow method is more robust than that of the triangular flow method and lognormal flow wave approximation (Figures 10–15). The results of personalized flow waveform method are the closest to one compared to the other methods, thus indicating a very good one to one correspondence. The personalized flow approximates the measured flow and gives better estimates of RM, RI, and PTT. The quantitative and objective comparison of the three flow wave methods is summarized in Table 2. To further strengthen the validity of the proposed method in obtaining the flow waveform from CAPW, we also calculated the RMSE between the actual known flow and the approximated flow using three methods (i.e., personalized flow, lognormal flow, and triangular flow). The proposed personalized flow method gave the smallest values (as shown in Table 2). The small errors indicate that the personalized flow wave shape is a good approximation for applying waveform analysis and improves wave separation analysis results compared to the other two methods.

## 4 Discussion

In this study, we applied a personalized wave to estimate the aortic flow waveform in two data sets (Nektar1D PWDB and Clinical data) to investigate the feasibility of CAPW separation. Moreover, the CAPW reflection indices calculated using the personalized estimated flow waveform were compared with the results derived from the traditional triangular flow wave and the recently proposed lognormal flow wave approximation method. The CAPW was decomposed into P_f_ and P_b_ using pressure-flow relations, and wave reflections were quantitatively and qualitatively analyzed. By experimental analysis, the correlation and consistency of the wave reflection indices calculated based on the personalized and measured flow waves are higher than the other two methods (Figures 10–15). From the perspective of RI, RM, and PTT, the RMSE between the personalized flow waveform and measured flow waveform are smaller than the difference between the other two methods (Table 2). Moreover, the shape of the personalized estimation flow wave is better than that of the triangle and lognormal flow waves (see Figure 8).

Also, the P_f_ and P_b_ of the CAPW decomposition by personalized flow waveforms are closer to the actual results. The errors of the amplitudes of P_f_ and P_b_ decomposed by the personalized estimated flow wave and CAPW are smaller (Table 2). The waveform of personalized flow is more consistent with the actual flow waveform compared with the lognormal and triangular flow waveform (Figure 8). Moreover, the biases between wave reflection indices calculated by decomposing CAPW with the measured and personalized flow are smaller. Furthermore, the P_f_ and P_b_ of the CAPW decomposition by personalized flow waveform are closer to the actual results in amplitude and waveform morphology than the other two methods (Nektar1D PWDB; RMSEs = 1.39 and 0.39, Table 2 and Clinical data; RMSEs = 3.29 and 1.37; Table 2). Using a triangle to estimate the flow waveform will lead to spikes, and also P_f_ and P_b_ calculated by triangle flow waves will also appear as spikes (see Figure 8). This will not happen in the measured flow, and the personalized flow is more reliable.

Through linear regression equation and Bland-Altman diagram analysis, RM, RI, and PTT obtained from personalized flow waveform are highly correlated with RM, RI, and PTT obtained from the measured flow (Figures 10–15). These show that the wave reflection indices can be calculated by the personalized estimated flow wave when the real flow wave is not convenient to measure. As shown in Figures 10–15, Bland–Altman plots generally revealed smaller biases and narrower 95% LOA (Limits of agreement) for the personalized flow waveform, compared with the triangular and lognormal flow waveform approximation. Wave reflection indices derived using the truly measured flow waveform and estimated flow waveforms using three methods are reported in Table 2. Based on the comparison of the results between the Nektar1D PWDB and clinical data, the Pearson correlation coefficients between the personalized flow wave, lognormal flow, triangular flow wave, and the measured flow wave indicate that the accuracy of the personalized flow wave is higher. It was notable that over the pulse wave reflection indices, the biases of RM, RI, and PTT were lower for the personalized flow waveform than the triangular and lognormal flow waveform in most cases, thus confirming the superior performance of the personalized flow method. In addition, compared with the triangle flow wave, the personalized flow wave is more consistent with the measured flow wave in terms of RI, RM, and PTT. Besides, the personalized flow wave method does not require complex statistical calculations like the lognormal approximation, nor does it need to establish a variance value in advance.

The clinical data used for validation in this paper are limited to young, healthy participants only, which is one of the limitations of this study. There was no vascular or cardiac disease model included in the 1-D model when generating the virtual subjects. The 1-D database also only represents healthy subjects to the limitation. The proposed method should be validated in different populations (i.e., patients with heart disease) further to investigate the generalizability of the personalized flow waveform method. In addition, it is feasible that PTT is estimated only by calculating the time delay of P_f_ and P_b_, but there is no comparison and correlation analysis with the measured carotid-femoral PTT and aortic pulse wave velocity. In order to better evaluate arterial stiffness, a comparison is necessary. The reliability of using the 30% ET as a surrogate of inflection point has not been rigorously proven, but it has just been used as a rule of thumb in previous studies. Typically, some degree of flow regurgitation occurs when the aortic valve closes, i.e., the actual aortic flow is negative at end-systole (shown in Figure 8). As with the triangular and the lognormal flow waves, the proposed personalized flow wave ignores this by setting the diastolic flow to 0 (Westerhof et al., 2006; Hao et al., 2022). Although the personalized flow wave improves the results of wave reflection and wave separation analysis compared to the other two methods, it is still necessary to further strengthen this research to implement this typical feature of aortic flow waveform. Furthermore, in early-systole, the flow peak obtained by the proposed personalized flow method is closer to the measured flow peak than the other methods, and occurs later in time compared to the measured waveform. Also, during the late-systolic part of the personalized flow waveform, the waveform overestimates the measured waveform (see Figure 8). There are still errors between the approximate personalized flow waveform and the measured flow waveform. Future research should focus on the three feature points (a, b, and c) involved in the Hermite interpolation operation in order to construct a flow wave that is more consistent with the measurement.

## 5 Conclusion

In this paper, a novel method of approximate estimation of flow waves based on the characteristics of the CAPW is proposed, and the feasibility of personalized flow separation in CAPW is evaluated. The results indicate that the personalized flow wave method generates more accurate aortic flow waveform. Experiments on Nektar1D PWDB and clinical data verify the feasibility of the proposed method. The personalized flow wave estimated by our proposed method is more consistent with the measured flow wave when used to calculate RM, RI, and PTT, compared to the triangle estimation and lognormal approximation. P_f_ and P_b_ decomposed from CAPW using personalized flow wave method have more accurate shapes and amplitudes than the other two methods. The personalized flow wave method improves CAPW separation results both in accuracy and reliability.

## Data Availability

The original contributions presented in the study are included in the article/supplementary material, further inquiries can be directed to the corresponding author.

## Abbreviations

CAPW, Central aortic pressure waveform; CVD, Cardiovascular disease; DBP, Diastolic blood pressure; ET, Ejection time; FDA, Food and Drug Administration; MAP, Mean arterial pressure; P_b_, Backward pressure wave; P_f_, Forward pressure wave; PTT, Pulse transit time; Q_b_, Back flow wave; Q_f_, Forward flow wave; RI, Reflection index; RM, Reflection magnitude; RMSE, Root mean square error; SBP, Systolic blood pressure; T_fb_, The time difference between P_f_ and P_b_; Z_c_, Characteristic impedance; Z_in_, Input impedance.
